# Ultraviolet B irradiation enhances the secretion of exosomes by human primary melanocytes and changes their exosomal miRNA profile

**DOI:** 10.1371/journal.pone.0237023

**Published:** 2020-08-12

**Authors:** Zeren Shen, Jiaqi Sun, Jinjin Shao, Jinghong Xu

**Affiliations:** 1 Department of Plastic Surgery, The First Affiliated Hospital, School of Medicine, Zhejiang University, Hangzhou, China; 2 College of Pharmaceutical Sciences, Zhejiang University, Hangzhou, China; Gustave Roussy, FRANCE

## Abstract

**Objective:**

Melanocytes play a central role in skin homeostasis. In this study, we focus on the function of melanocyte releasing exosomes as well as exosomal microRNAs (miRNAs) and investigate whether ultraviolet B (UVB) irradiation exerts an impact on it.

**Materials and methods:**

Exosomes derived from human primary melanocytes were isolated through differential centrifugation and were identified in three ways, including transmission electron microscopy observation, nanoparticle tracking analysis, and Western blot analysis. Melanocytes were irradiated with UVB for the indicated time, and then melanin production and exosome secretion were measured. The exosomal miRNA expression profile of melanocytes were obtained by miRNA sequencing and confirmed by real-time PCR.

**Results:**

Exosomes derived from human primary melanocytes were verified. UVB irradiation induced melanin production and increased the exosome release by the melanocytes. In total, 15 miRNAs showed higher levels in UVB-irradiated melanocyte-derived exosomes compared with non-irradiated ones, and the top three upregulated exosomal miRNAs were miR-4488, miR-320d, and miR-7704 (fold change > 4.0).

**Conclusion:**

It is verified for the first time that UVB irradiation enhanced the secretion of exosomes by melanocytes and changed their exosomal miRNA profile. This findings open a new direction for investigating the communication between melanocytes and other skin cells, and the connection between UVB and skin malignant initiation.

## 1. Introduction

In the epidermis, which is the outermost thin layer of the skin, melanocytes move about to inject melanin into keratinocytes; moreover, they are in contact with Langerhans cells, with fibroblasts, with sensory neurons through their cutaneous axon terminals, and with endothelial cells [1–3]. Cells communicate either via secreted soluble factors or via extracellular vesicles (EVs) [4,5]. Exosomes are common EVs originated form endosomes with a diameter of 30–150 nm [6,7], and they carry numerous biological substances, such as proteins, mRNAs, microRNAs (miRNAs), cytokines, and transcription factors [8]. Studies have shown that melanocytes are the target of exosomes secreted by other cells, such as keratinocytes [9–11]; as for exosomes derived from melanocytes, their role in other cells is rarely investigated. Solar radiation stimulates the synthesis of melanin in melanocytes and the transport of melanin-containing melanosomes to neighboring epidermal cells, resulting in skin pigmentation. In addition to facilitating pigmentation, melanocytes possess an important defense mechanism to provide protection against ultraviolet (UV)-induced oxidative and genotoxic stresses, wherein they amplify and send signals to other cells within an organized regulatory network to maintain skin homeostasis [12]. However, excessive UVB will destroy the repair mechanism of melanocytes, even leading to skin cancer. In this study, we focus on the function of melanocyte releasing exosomes as well as exosomal microRNAs (miRNAs), and investigate whether UVB irradiation exerts an impact on it and ultimately on intercellular communication and skin malignant initiation.

## 2. Materials and methods

### 2.1. Antibodies and other reagents

Rabbit polyclonal anti-TSG101 (14497-1-AP; 1:1000), rabbit polyclonal anti-HSP70 (10995-1-AP; 1:1000), and rabbit polyclonal anti-MITF (Proteintech1092-1-AP; 1:1000) were obtained from Proteintech (China). Rabbit polyclonal anti-CD63 (ab118307; 1:1000) and rabbit polyclonal anti-Tyrosinase (ab170905; 1:1000) were procured from Abcam (England). Rabbit polyclonal anti-GAPDH (SB100242-T40; 1:1000) and rabbit polyclonal anti-Calnexin (CST2679; 1:1000) were obtained from Sino Biological (China) and CST (USA), respectively. Horseradish peroxidase (HRP)-conjugated goat anti-rabbit IgG (A#21020; 1:5000) and horseradish peroxidase (HRP)-conjugated goat anti-mouse IgG (A#21010; 1:5000) were obtained from Abbkine (USA). Mouse polyclonal anti-CD9 (1:1000) antibody was donated by Yuan Gao.

### 2.2. Cell culture and UVB treatments

Adult human epidermal melanocytes (HEMs) were obtained from Sciencell (USA). The HEMs were grown in Melanocyte Medium, which contained melanocyte growth factors (Sciencell #2201), at 37°C and 5% CO_2_. Only melanocytes from the second to fifth passage were used. The melanocytes were irradiated with UVB at doses of 30 mJ/cm^2^ or 60 mJ/cm^2^ one time each at a 24-h interval for three consecutive days and then cultivated for 3 days (for exosome isolation) or 2 weeks (for melanin assay). Before irradiation, the medium was replaced with phosphate buffered saline (PBS), and irradiation was delivered by a UVB lamp (290–320 nm) (Philips TL 20W/12); subsequently, PBS was immediately removed and then replaced with complete medium.

### 2.3. Melanin assay

Melanocytes were lysed by using RIPA lysis buffer (0.1 M Tris-HCl pH 7.2, 1% NP-40, 0.01% SDS and protease-inhibitor cocktail) and centrifuged at 15,000 g for 10 min. The supernatant containing proteins was used in protein quantification with a bicinchoninic acid (BCA) kit (Thermo Fisher Scientific, Waltham, MA, USA), and the pellets containing melanin were dissolved in 1 N NaOH (added with 10% DMSO) and incubated for 30 min at 60°C. Protein and melanin contents were determined by measuring the absorbance at 562 and 450 nm, respectively.

### 2.4. Exosome isolation

The medium was replaced with fetal bovine serum free culture medium 3 days before we collected the medium once for exosome isolation. Exosomes were isolated using differential centrifugation, as follows: The media were centrifuged at 300 g and 3,000 g for 15 min at 4°C, and the precipitates were removed. The supernatant was then centrifuged at 10,000 g for 30 min at 4°C, and the precipitates were again removed. Subsequently, the supernatant was filtered by passing through a vacuum-connected 0.22 μm filter. The supernatant was again centrifuged at 120,000 g for 30 min at 4°C. The precipitates were collected, resuspended, and then washed in PBS and centrifuged at 120,000 g for 30 min at 4°C, and then the precipitates were collected. The exosomes were resuspended in PBS and stored at −80°C until further use.

### 2.5. Transmission electron microscopy

The exosomes were fixed in 4% glutaraldehyde overnight at 4°C. A sample (10 μL) was dripped and precipitated on a copper net for 1 min; the floating liquid was absorbed. Similarly, hydrogen peroxide acetate (2%, 10 μL) was dripped and precipitated on the copper net for 1 min. The floating liquid was absorbed, allowed to dry at room temperature for several minutes, and then examined under an electron microscope.

### 2.6. Nanoparticle tracking analysis (NTA)

The isolated exosomes were diluted in PBS (1:10) for the measurement of particle size and concentration. Size distribution was directly determined using qNano (Izon Sciences Ltd, NZ).

### 2.7. Western blot analysis

Cells or exosomes were lysed on ice in a lysis buffer with a protease inhibitor cocktail. The cell lysates or exosomes were incubated in a loading buffer and then boiled for 10 min. Total protein was extracted, and the protein concentration was assayed using a BCA kit (Thermo Fisher Scientific, Waltham, MA, USA). Following polyacrylamide gel electrophoresis for 35 min at 80 V and for 45 min at 120 V, protein was transferred onto nitrocellulose membranes (Millipore, USA). The membranes were blocked in PBS-Tween 20 (PBST) (PBS buffer containing 0.1% Tween-20) with 5% non-fat milk for 1 h at room temperature. Subsequently, the membrane was incubated with the indicated primary antibody overnight at 4°C. The membrane was washed three times with PBST for 10 min for each round and then incubated in HRP-conjugated secondary antibody for 1 h. Finally, blots were developed by using the ECL and then scanned under an optical luminescence instrument. Three parallel experiments were performed.

### 2.8. Real-time PCR

The total cellular RNA was extracted using a Total RNA Kit II (EC Science, China) and was reverse-transcribed into cDNA using a reverse transcription kit (TAKARA, Japan). The PCR system was prepared according to the instruction provided in the SYBR qPCR Mix kit (TAKARA, Japan). Variance in the expression levels of microphthalmia-associated transcription factor (MITF), TYR, and miRNAs was detected using a real-time PCR instrument (Bio-Rad), with GAPDH (for mRNA) and U6 (for miRNA) as the internal references; ΔCT = 2^−ΔΔCt^.

### 2.9. Small RNA sequencing and data analysis

Exosomal RNA was extracted by HiPure Liquid miRNA Kit/HiPure Serum/Plasma miRNA Kit (Megan, China). The quantity and integrity of exosomal RNA yield was assessed by using the Qubit®2.0 (Life Technologies, USA) and Agilent 2200 TapeStation (Agilent Technologies, USA) separately. 50 ng exosomal RNA of each samples were used to prepare small RNA libraries by NEBNext® Multiplex Small RNA Library Prep Set for Illumina (NEB, USA) according to manufacturer’s instructions. The libraries were sequenced by HiSeq 2500 (Illumina, USA) with single-end 50 bp at Ribobio Co. Ltd (Ribobio, China).

The raw reads were processed by filtering out containing adapter, poly “N,” low quality, smaller than 17nt reads by FASTQC to get clean reads. Mapping reads were obtained by mapping clean reads to reference genome of by BWA. MiRDeep2 was used to identify known mature miRNA based on miRBase21 (www.miRBase.org) and predict novel miRNA. The expression levels of miRNAs were normalized by RPM, RPM is equal to (number of reads mapping to miRNA/ number of reads in Clean data)×10^6^. Differential expressions between two sets of samples were calculated using the DESeq2 algorithm, according to the criteria of |log2(Fold Change)| ≥ 1 and P-value < 0.05. In total, 636 miRNAs were identified between the UVB-irradiated melanocyte-derived exosome and the non-irradiated melanocyte-derived exosome groups, including 39 differentially expressed miRNAs (Fig 3A and S1 Table). The miRNA sequencing data has been uploaded to Gene Expression Omnibus (GEO) (record number: GSE152251). TargetScan, miRDB, miRTarBase and miRWalk were used to predict target genes of selected miRNAs. KOBAS was used for further network analysis.

## 3.0. Statistical analysis

Statistical analysis was conducted using the SPSS 21.0 software (IBM, Armonk, NY, USA). Student’s t-test was used to determine the statistical significance of the differences in melanin expression in the irradiated and non-irradiated cells and in the amount of exosomes released in both groups.

## 3. Results

### 3.1. Characterization of primary melanocyte-derived exosomes

**Fig 1:**
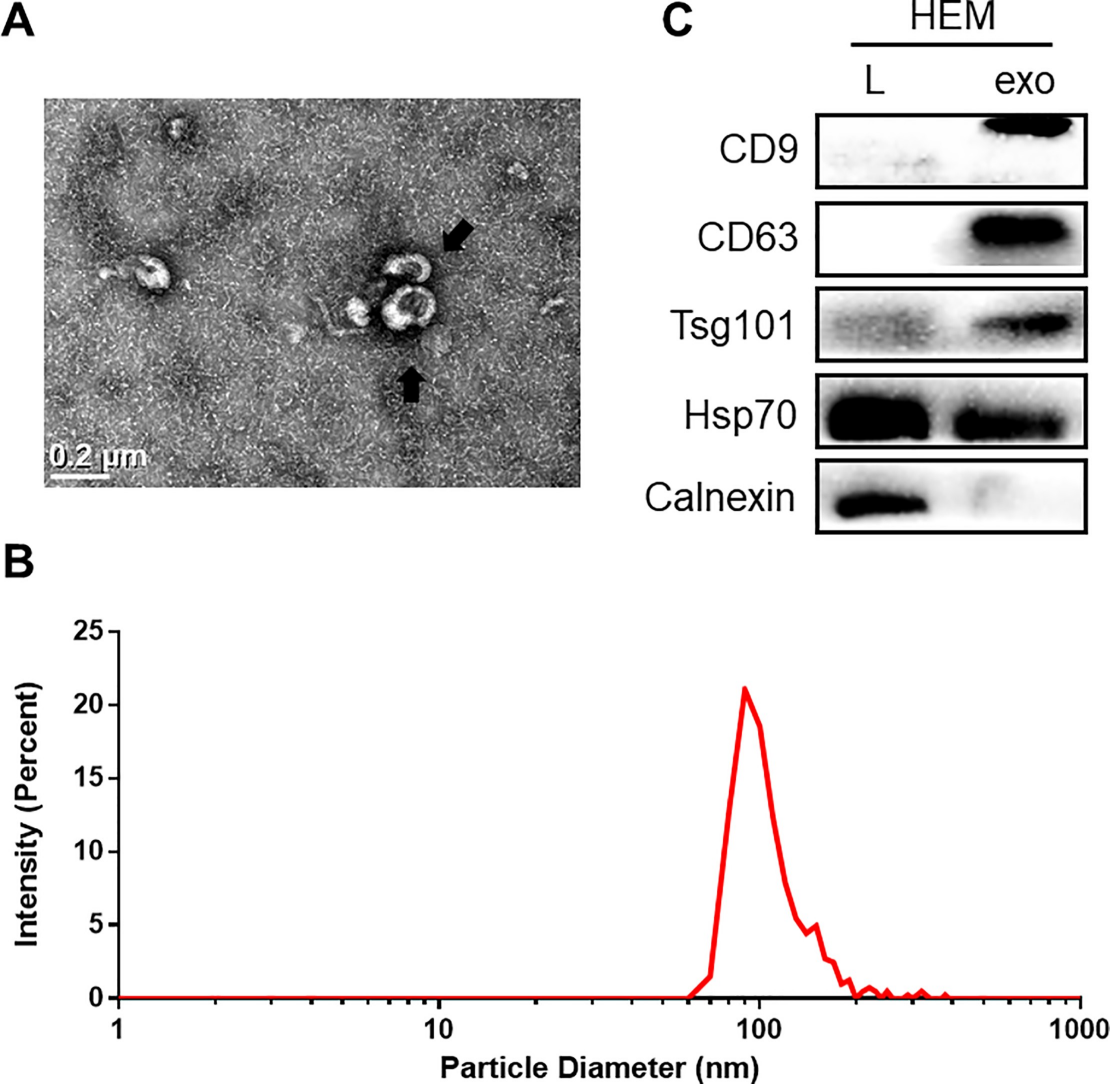
Characterization of primary melanocyte-derived exosomes. (A) The collected products had a cup shape as evidenced by using a transmission electron microscope. The arrow indicates exosomes, and the scale bar represents 0.2 μm. (B) Size distribution of microvesicles (nm) assessed by nanoparticle tracking analysis. (C) Confirmation of exosome specific markers such as CD9, CD63, TSG101. Exosomal negative maker Calnexin was lost in the samples.

We isolated exosomes from the supernatant of human primary melanocytes through differential ultracentrifugation. We identified the particles we isolated according to the characteristics of the exosomes [13]. First, transmission electron microscopy showed that the isolated particles had a cup-shaped canonical exosome morphology with a double-layer membrane structure (Fig 1A). Second, the NTA results indicated that the diameters of the isolated particles were 80–160 nm, with a mean diameter of 120 nm (Fig 1B). Western blot analysis revealed that the particles expressed the molecular markers widely recognized for exosomes, such as CD63, CD9, TSG101, and HSP70 (Fig 1C). Moreover, the non-exosomal marker (Calnexin) was absent in the exosome‐enriched fraction (Fig 1C). Therefore, the particles we isolated from the supernatant of primary melanocytes were indeed exosomes.

### 3.2. Effect of UVB on the secretion of melanin and exosomes by primary melanocytes

**Fig 2:**
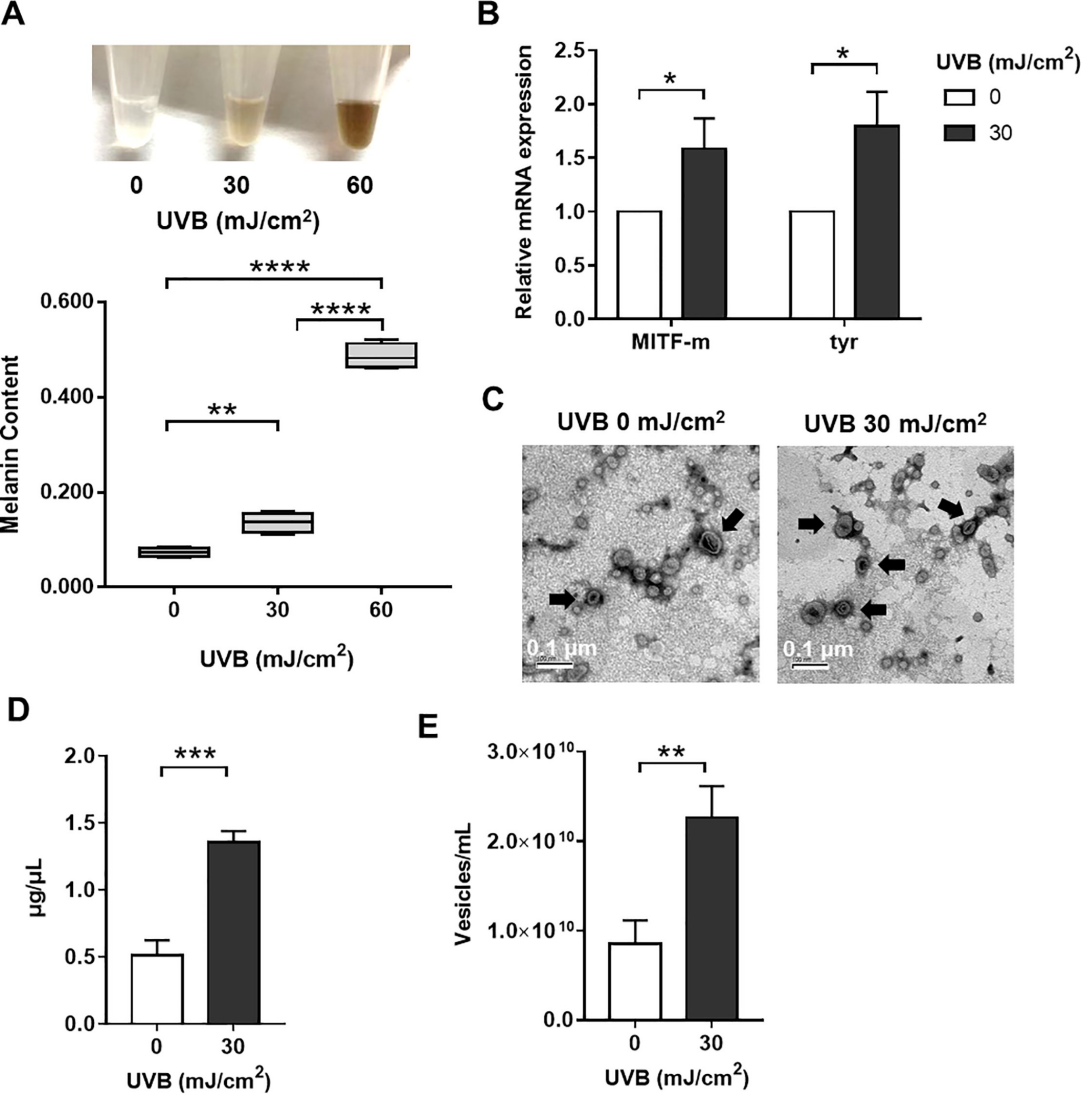
Effect of UVB on the production of melanin and secretion of exosomes by primary melanocytes. (A) Melanocytes irradiated by various doses of UVB and the color of the cell lysis buffer was monitored. The melanin content was measured at 450 nm. The data are representative of three independent experiments (***, P < 0.005; ****, P < 0.0 001). (B) Expression of tyrosinase (tyr) and MITF at the mRNA level was determined by using qRT-PCR (*, P < 0.05). (C) Electron micrographs showed the cup‐shaped vesicles. The arrow indicates exosomes, and the scale bar represents 0.2 μm. Exosomes were then quantified with (D) BCA assay and (E) nanoparticle tracking analysis. (**, P < 0.001; ***, P < 0.0 01).

To investigate whether UVB could induce melanocyte pigmentation, we treated the melanocytes with 30 mJ/cm^2^ UVB radiation for three consecutive days. The melanin assay results showed that UVB irradiation promoted the production of melanin by the melanocytes in a UVB dose-dependent manner (Fig 2A). Moreover, the results of qRT-PCR revealed the significantly upregulated expression levels of MITF, which is a master transcriptional melanogenesis regulator, and of tyrosinase (TYR) [14,15], the key enzyme in melanin biosynthesis (Fig 2B) [16]. Taken together, the results suggested that UVB acts on TYR and MITF to increase melanocyte pigmentation.

Moreover, after evaluating the particle density using electron microscopy (Fig 2C) and NTA (Fig 2E) and after assessing the total protein concentration of exosomes by using a BCA kit (Fig 2D), we found that the release of exosomes from melanocytes was significant increased.

### 3.3. Effect of UVB on the exosomal miRNAs expression of primary melanocytes

**Fig 3:**
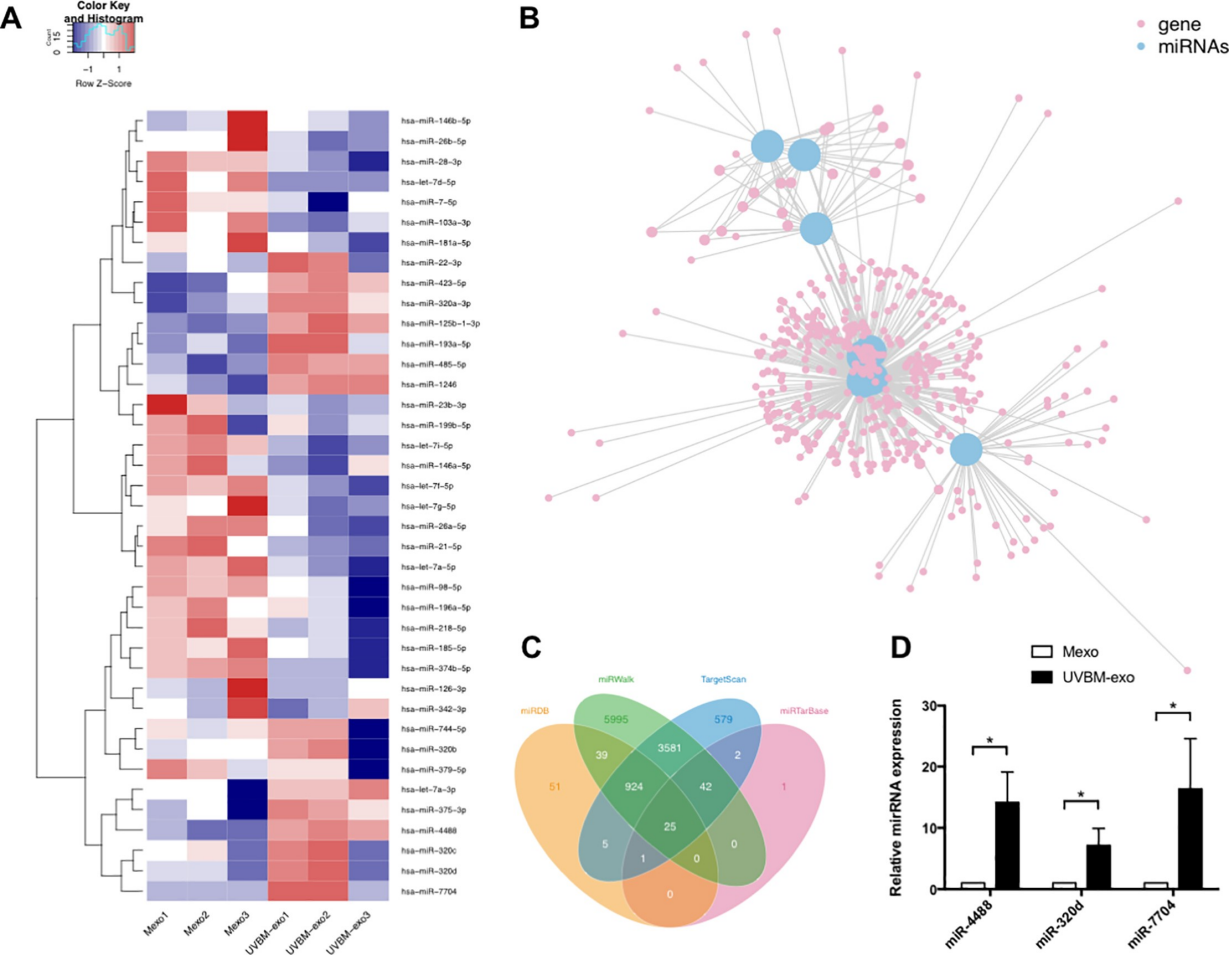
The expression levels of identified microRNAs (miRNAs) in UVB-irradiated melanocyte-derived exosomes (UVBM-exo group) and non-irradiated melanocyte-derived exosomes (Mexo group). (A) Heat map representation of small RNA sequencing data about the expression levels of identified miRNAs in UVBM-exo and Mexo groups. The data are from three independent experiments in each group. The melanocytes in group of UVBM-exo were treated with 30 mJ/cm^2^ UVB radiation for three consecutive days. The upregulated miRNAs are depicted on shades of red; downregulated miRNAs are in shades of blue. (B) Network analysis of 8 upregulated miRNAs and their 497 target genes by KOBAS. These 8 upregulated miRNAs include miR-4488, miR-1246, miR-423-5p, miR-320c, miR-320d, miR-320b, miR-125b-1-3p, and miR-193a-5p (see S2 Table for specific data). Blue dots represent the upregulated miRNAs and pink dots represent their target genes. (C) Number of target genes of miR-320d predicted by TargetScan, miRDB, miRTarBase and miRWalk. Yellow area represents miRDB database, green represents miRWalk, blue represents TargetScan, and pink represents miRTarBase. The four databases together predict 25 target genes (at the center of the diagram) of miR-320d. (D) Expression of miR-4488, miR-320d, and miR-7704 was determined by using qRT-PCR (*, P < 0.05). The data are from three independent experiments in each group.

Further, we explored expression changes in melanocyte-derived exosomal miRNA profile before and after UVB irradiation by miRNA sequencing. As shown in Fig 3A, there were 15 exosomal miRNAs of melanocytes were upregulated after UVB irradiation, including miR-4488, miR-1246, miR-423-5p, miR-320a-3p, miR-320c, miR-320d, miR-320b, miR-375-3p, miR-125b-1-3p, miR-193a-5p, miR-485-5p, miR-7704, let-7a-3p, miR-22-3p, and miR-744-5p (fold change > 1.0). The top three upregulated miRNAs included miR-4488, miR-320d, and miR-7704, which were significantly elevated in UVB-irradiated melanocyte-derived exosomes compared with non-irradiated ones (fold change > 4.0). Network analysis showed 497 target genes of upregulated miRNAs in total, including 25 of miR-320d, 8 of miR-4488, and 0 of miR-7704 (Fig 3B, 3C and S2 Table). Genes involved in autophagy, DNA damage and repair, and oncogenesis, such as MRPS18B, ULK1, MEN1, and TPD52, were predicted to be targeted by selected miRNAs.

Next, we decided to validate the miRNA sequencing data by qRT-PCR on these top three upregulated miRNAs. The same experiment was performed on the treatment and the control groups under the same experimental conditions used in the miRNA sequencing. Results confirmed that miR-4488, miR-320d, and miR-7704 were strongly increased in UVB-irradiated melanocyte-derived exosomes (Fig 3D). We also performed qRT-PCR to compare the change between expression of miR-4488, miR-320d, and miR-7704 in UVB-irradiated melanocytes and in non-irradiated melanocytes (S1 Fig). None of the three miRNAs showed an up-regulation in cells. The expression level of miR-320d and miR-7704 even decreased in UVB-irradiated melanocytes compared with non-irradiated melanocytes. This may indicate that UVB promotes the secretion of miRNAs by melanocytes specifically through exosomes.

## 4. Discussion

In this study, we enriched and verified the exosomes derived from human primary melanocytes. Moreover, we determined the secretion ability and the secreted exosomal miRNAs expression patterns of the UVB-irradiated melanocytes.

Melanocytes are dendritic, neural crest-derived cells that are generally located in the basal layer of the epidermis, but they can also be found in hair follicles, choroid, cochlea, brain, and heart [17–19]. Highlighting the unique properties of this cell type, it is well-known but underestimated to synthesize and secrete melanin. In the epidermis, a melanocyte is attached through its dendrites to dozens of keratinocytes and then transfers melanin-containing melanosomes to these cells; this network of cells is called an epidermal melanin unit [20]. Researches have demonstrated that melanocytes interact with Langerhans cell, with peripheral neurons through their cutaneous axon terminals, and with endothelial cells, which are involved in skin immune response, sensing, and vascularization, respectively [3,21–23]. Melanocytes can even enter the dermis and interact with fibroblasts [24–26]. Moreover, melanocytes respond to paracrine factors, which can more effectively repair DNA damage and fight oxidative stress [27,28]. Melanocytes, which play a vital role in the skin, are capable of receiving signals from biological, physical, and chemical environments; consequently, they adjust their inner state and send signals both to their adjacent and distant tissue environments [24].

UV irradiation is the most important physical factor affecting melanocytes, which can also cause a considerable fraction of skin cancers [29]. The right amount of UV, typically UVB radiation (290–320 nm), can promote mineral metabolism and vitamin D formation; however, excessive and long-term irradiation acts as oxidative and genotoxic stress, which may induce thymidine breaks in DNA as well as promote apoptosis, inflammation, immune cell activation, secretion of various cytokines and chemokines, and may even cause skin cancer [29,30]. To repair UVB radiation-induced damages, cells tend to alter the expression of genes particularly those involved in stress response, as well as control cell cycle, DNA synthesis, and DNA repair [31,32]. UVB can activate melanocytes to produce pro-opiomelanocortin (POMC) and cytokines, such as α-melanocyte-stimulating hormone (α-MSH), adrenocorticotrophic hormone, basic fibroblast growth factor (bFGF) to regulate melanogenesis and other stress responses [33]. Acting as epithelial “stress sensors,” melanocytes not only defend themselves against UV damage, but they also protect other cells in the skin. Although melanogenesis is the primary defense mechanism of melanocytes against environmental stresses, it is certainly not the only defense mechanism that exists in melanocytes and perhaps not even the most important one [24]. Keratinocytes, fibroblasts, and Langerhans cells have been reported to actively interact with the melanocytes and influence their functioning. However, few studies have focused on the effects of melanocytes on other cells.

Cells communicate either via secreted soluble factors or via EVs [4,5]. Melanocytes can release EVs, and researches have used mRNAs or miRNAs contained in exosomes derived from primary melanocytes as a reference to study the contents of melanoma cell-derived exosomes [34,35]. There are relatively few studies that have focused on the effects of melanocytes on other cells. Thus, we focused on the ability of melanocytes to secrete EVs to influence other cells, and we have successfully enriched the melanocyte-derived exosomes and verified them. The technical procedures used to isolate, purify, and quantify exosomes secreted by the melanocytes are a crucial point to consider for data standardization. Our results further expanded the characterization of EVs secreted by melanocyte; the vesicles isolated from the cell culture supernatants of melanocytes express proteins present in the intraluminal vesicles (ILVs) of multivesicular bodies (MVBs) (CD63, CD9, and TSG101). The vesicles were typically cup-shaped as revealed by electron microscopy and with diameters ranging from 80 nm to 160 nm as assessed by NTA. In our study, we found for the first time that UVB irradiation could enhance the secretory function of melanocytes and change their exosomal miRNA profile, pointing to the cellular response to irradiation, thereby enhancing the communication between melanocytes and other cells.

Circulating miRNAs play an important role in the multiple intercellular communication pathways, and a large fraction of these are found in exosomes [36]. Thus, we explored expression changes in melanocyte-derived exosomal miRNA profile before and after UVB irradiation, and observed specific patterns of exosomal miRNA response to UVB irradiation. In total, 15 miRNAs showed higher levels in UVB-irradiated melanocyte-derived exosomes compared with non-irradiated ones. Upregulated miRNAs may result in the dysfunction of target genes involved in control cell cycle, autophagy, DNA damage and repair, and oncogenesis. Among the top three upregulated miRNAs in UVB-irradiated melanocyte-derived exosomes, miR-4488, miR-320d, and miR-7704 have all been reported to be involved in human cancers, and miR-4488 in melanoma, in particular. In recent publications, miR-4488 was reported to be upregulated in BRAFi-resistant melanoma cell lines [37] and in melanoma-drived exosomes [38]. In addition, exosomal miR-320d has been proposed as a serum biomarker for metastatic colorectal cancer [39]. The miR-7704 from extracellular vesicles showed the most pro-tumorigenic alterations in Erlotinib-resistant head and neck squamous cell carcinoma [40]. Thus, we proposed that the changed miRNA profile in exosomes following UVB irradiation may have a close connection with the initiation of the malignant skin diseases. By using TargetScan, miRDB, miRTarBase, and miRWalk to predicted targets of miR-4488, miR-320d, and miR-7704, MRPS18B is related to DNA damage and repair, ULK1 is related to autophagy, and MEN1 and TPD52 are related to oncogenesis. A deeper assessment of the role of miR-4488, miR-320d, and miR-7704 upregulation in the initiation of skin malignance is currently under investigation.

**Fig 4:**
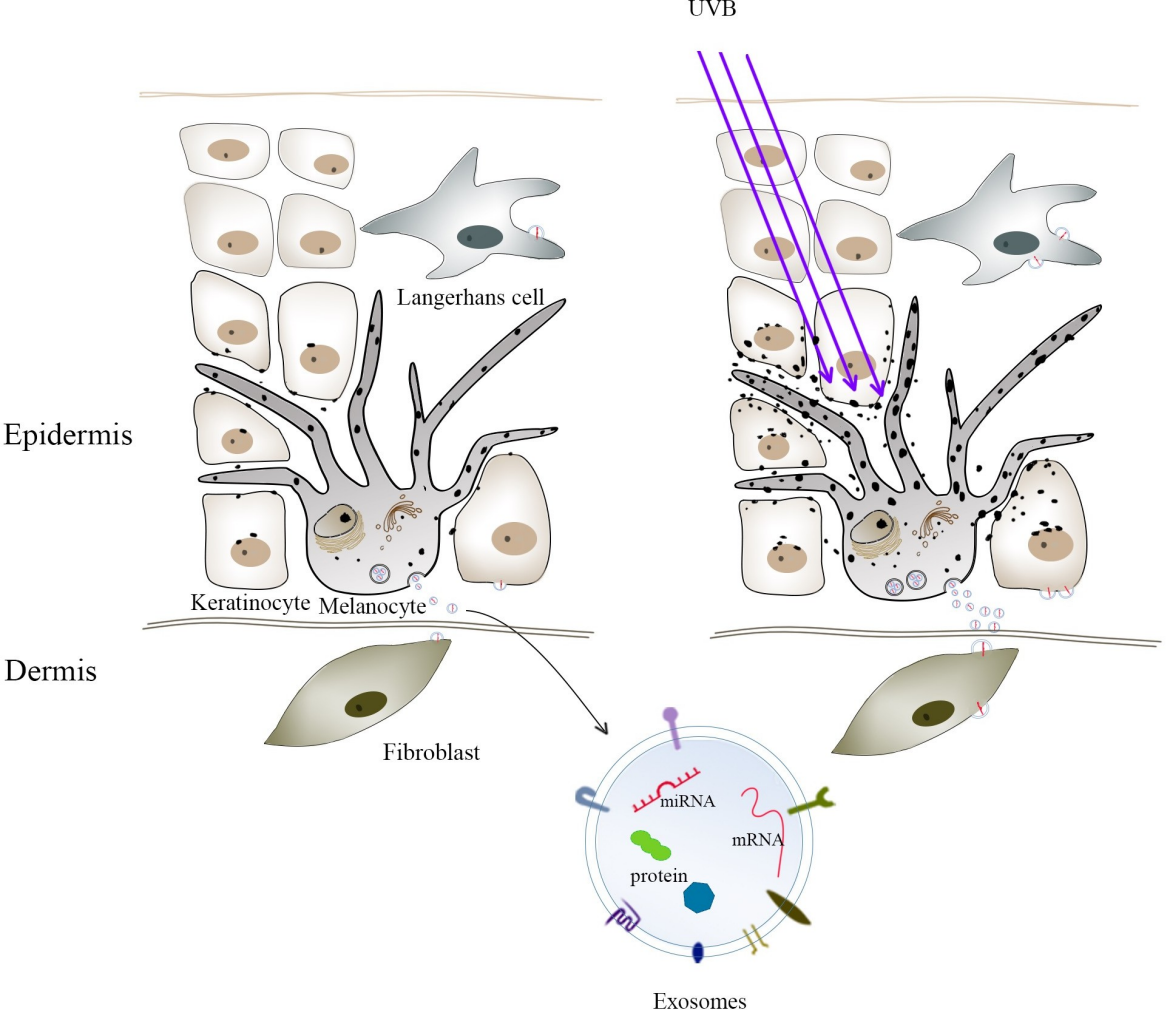
Exosome-mediated crosstalk between melanocytes and other skin cell types, such as keratinocytes, Langerhans cells, and fibroblasts. UVB irradiation enhanced the secretion of exosomes by melanocytes, thereby enhancing the communication between melanocytes and other cells.

Considering the multiple intercellular communication pathways between melanocytes and other cells, the contents of melanocyte-derived exosomes may play an important role in the interaction of melanocytes with the exosome recipient cells. Fig 4 proposes an exosome-mediated crosstalk between melanocytes and other skin cell types. Such interactions can affect a number of key cellular signaling pathways that modulate essential cell functions, such as skin pigmentation, vascularization, sensing, and immune response. Thus, investigating the role of melanocytes-derived exosomes as signaling vesicles that are potentially involved in skin homeostasis may be an important step in the attempt to design strategies for the efficient treatment of several skin disorders.

The main limitation of this pilot study is that it only provides preliminary data on melanocytes releasing exosomes as well as exosomal miRNAs and being affected by UVB irradiation. The exact function of all the changes in this response remains unclear. The effect of melanocyte-derived exosomes containing different signal molecules on other cells deserves further study. More attention should be devoted on the functional analysis of changes in miRNA expression in response to UVB. Nonetheless, we are eager to share our findings with our peers, and we look forward to further explorations in this area. This re-assessment of melanocyte biology may provide a novel direction in studying various cell-to-cell communication pathways among skin cells, and findings will hopefully provide opportunities for the prevention and treatment of skin diseases.

## Supporting information

S1 Raw images Raw images of Western blot (for Fig 1C). (PDF)

S1 Fig The expression levels of identified miRNAs in UVB-irradiated melanocytes (UVBM group) and non-irradiated melanocytes (M group) determined by using qRT-PCR. The data are from three independent experiments in each group (*, P < 0.05).
(PDF)

S1 Table Differentially expressed miRNAs in UVB-irradiated melanocyte-derived exosomes (UVBM-exo group) and non-irradiated melanocyte-derived exosomes (Mexo group) determined by using miRNA sequencing. (XLS)

S2 Table Network connection of upregulated miRNAs and target genes. (XLSX)

## References

[1] Van Den Bossche K, Naeyaert JM, Lambert J. The quest for the mechanism of melanin transfer. Traffic. 2006;7(7):769–78. 10.1111/j.1600-0854.2006.00425.x 16787393

[2] Lewis JM, Burgler CD, Freudzon M, Golubets K, Gibson JF, Filler RB, et al Langerhans Cells Facilitate UVB-Induced Epidermal Carcinogenesis. J Invest Dermatol. 2015;135(11):2824–33. 10.1038/jid.2015.207 26053049 PMC4640962

[3] Nordlund JJ. The Medical Treatment of Vitiligo: An Historical Review. Dermatol Clin. 2017;35(2):107–16. 10.1016/j.det.2016.11.001 28317520

[4] Gyorgy B, Szabo TG, Pasztoi M, Pal Z, Misjak P, Aradi B, et al Membrane vesicles, current state-of-the-art: emerging role of extracellular vesicles. Cell Mol Life Sci. 2011;68(16):2667–88. 10.1007/s00018-011-0689-3 21560073 PMC3142546

[5] Cocucci E, Racchetti G, Meldolesi J. Shedding microvesicles: artefacts no more. Trends Cell Biol. 2009;19(2):43–51. 10.1016/j.tcb.2008.11.003 19144520

[6] Simpson RJ, Jensen SS, Lim JW. Proteomic profiling of exosomes: current perspectives. Proteomics. 2008;8(19):4083–99. 10.1002/pmic.200800109 18780348

[7] Raposo G, Stoorvogel W. Extracellular vesicles: exosomes, microvesicles, and friends. J Cell Biol. 2013;200(4):373–83. 10.1083/jcb.201211138 23420871 PMC3575529

[8] Chevillet JR, Kang Q, Ruf IK, Briggs HA, Vojtech LN, Hughes SM, et al Quantitative and stoichiometric analysis of the microRNA content of exosomes. Proc Natl Acad Sci U S A. 2014;111(41):14888–93. 10.1073/pnas.1408301111 25267620 PMC4205618

[9] Lo Cicero A, Delevoye C, Gilles-Marsens F, Loew D, Dingli F, Guere C, et al Exosomes released by keratinocytes modulate melanocyte pigmentation. Nat Commun. 2015;67506. 10.1038/ncomms8506 PMC4491833 26103923

[10] Liu Y, Xue L, Gao H, Chang L, Yu X, Zhu Z, et al Exosomal miRNA derived from keratinocytes regulates pigmentation in melanocytes. J Dermatol Sci. 2019;93(3):159–67. 10.1016/j.jdermsci.2019.02.001 30904353

[11] Kim NH, Choi SH, Kim CH, Lee CH, Lee TR, Lee AY. Reduced MiR-675 in exosome in H19 RNA-related melanogenesis via MITF as a direct target. J Invest Dermatol. 2014;134(4):1075–82. 10.1038/jid.2013.478 24335901

[12] Slominski A, Paus R, Schadendorf D. Melanocytes as "sensory" and regulatory cells in the epidermis. J Theor Biol. 1993;164(1):103–20. 10.1006/jtbi.1993.1142 8264240

[13] Mincheva-Nilsson L, Baranov V, Nagaeva O, Dehlin E. Isolation and Characterization of Exosomes from Cultures of Tissue Explants and Cell Lines. Curr Protoc Immunol. 2016;11514.42.1–21. 10.1002/cpim.17 27801511

[14] Cheli Y, Ohanna M, Ballotti R, Bertolotto C. Fifteen-year quest for microphthalmia-associated transcription factor target genes. Pigment Cell Melanoma Res. 2010;23(1):27–40. 10.1111/j.1755-148X.2009.00653.x 19995375

[15] Shibahara S, Yasumoto K, Amae S, Udono T, Watanabe K, Saito H, et al Regulation of pigment cell-specific gene expression by MITF. Pigment Cell Res. 2000;13 Suppl 898–102. 10.1034/j.1600-0749.13.s8.18.x 11041365

[16] Hearing VJ, Tsukamoto K. Enzymatic control of pigmentation in mammals. Faseb j. 1991;5(14):2902–9. 1752358

[17] Goldgeier MH, Klein LE, Klein-Angerer S, Moellmann G, Nordlund JJ. The distribution of melanocytes in the leptomeninges of the human brain. J Invest Dermatol. 1984;82(3):235–8. 10.1111/1523-1747.ep12260111 6699426

[18] Yajima I, Larue L. The location of heart melanocytes is specified and the level of pigmentation in the heart may correlate with coat color. Pigment Cell Melanoma Res. 2008;21(4):471–6. 10.1111/j.1755-148X.2008.00483.x 18627529

[19] Mjaatvedt CH, Kern CB, Norris RA, Fairey S, Cave CL. Normal distribution of melanocytes in the mouse heart. Anat Rec A Discov Mol Cell Evol Biol. 2005;285(2):748–57. 10.1002/ar.a.20210 15977222

[20] Fitzpatrick TB, Breathnach AS. [THE EPIDERMAL MELANIN UNIT SYSTEM]. Dermatol Wochenschr. 1963;147481–9. 14172128

[21] Kim EH, Kim YC, Lee ES, Kang HY. The vascular characteristics of melasma. J Dermatol Sci. 2007;46(2):111–6. 10.1016/j.jdermsci.2007.01.009 17363223

[22] Kim EJ, Park HY, Yaar M, Gilchrest BA. Modulation of vascular endothelial growth factor receptors in melanocytes. Exp Dermatol. 2005;14(8):625–33. 10.1111/j.0906-6705.2005.00345.x 16026585

[23] Carrasco E, Soto-Heredero G, Mittelbrunn M. The Role of Extracellular Vesicles in Cutaneous Remodeling and Hair Follicle Dynamics. Int J Mol Sci. 2019;20(11). 10.3390/ijms20112758 PMC6600598 31195626

[24] Plonka PM, Passeron T, Brenner M, Tobin DJ, Shibahara S, Thomas A, et al What are melanocytes really doing all day long…? Exp Dermatol. 2009;18(9):799–819. 10.1111/j.1600-0625.2009.00912.x 19659579 PMC2792575

[25] Rendl M, Lewis L, Fuchs E. Molecular dissection of mesenchymal-epithelial interactions in the hair follicle. PLoS Biol. 2005;3(11):e331 10.1371/journal.pbio.0030331 16162033 PMC1216328

[26] Schneider MR, Schmidt-Ullrich R, Paus R. The hair follicle as a dynamic miniorgan. Curr Biol. 2009;19(3):R132–42. 10.1016/j.cub.2008.12.005 19211055

[27] Kupper TS, Chua AO, Flood P, McGuire J, Gubler U. Interleukin 1 gene expression in cultured human keratinocytes is augmented by ultraviolet irradiation. J Clin Invest. 1987;80(2):430–6. 10.1172/JCI113090 3497177 PMC442255

[28] Wakamatsu K, Graham A, Cook D, Thody AJ. Characterisation of ACTH peptides in human skin and their activation of the melanocortin-1 receptor. Pigment Cell Res. 1997;10(5):288–97. 10.1111/j.1600-0749.1997.tb00688.x 9359624

[29] Lin JY, Fisher DE. Melanocyte biology and skin pigmentation. Nature. 2007;445(7130):843–50. 10.1038/nature05660 17314970

[30] Zbytek B, Wortsman J, Slominski A. Characterization of a ultraviolet B-induced corticotropin-releasing hormone-proopiomelanocortin system in human melanocytes. Mol Endocrinol. 2006;20(10):2539–47. 10.1210/me.2006-0116 16740657 PMC1847418

[31] Marrot L, Meunier JR. Skin DNA photodamage and its biological consequences. J Am Acad Dermatol. 2008;58(5 Suppl 2):S139–48. 10.1016/j.jaad.2007.12.007 18410800

[32] Dziunycz P, Iotzova-Weiss G, Eloranta JJ, Lauchli S, Hafner J, French LE, et al Squamous cell carcinoma of the skin shows a distinct microRNA profile modulated by UV radiation. J Invest Dermatol. 2010;130(11):2686–9. 10.1038/jid.2010.169 20574436

[33] Kauser S, Schallreuter KU, Thody AJ, Gummer C, Tobin DJ. Regulation of human epidermal melanocyte biology by beta-endorphin. J Invest Dermatol. 2003;120(6):1073–80. 10.1046/j.1523-1747.2003.12242.x 12787137

[34] Bardi GT, Al-Rayan N, Richie JL, Yaddanapudi K, Hood JL. Detection of Inflammation-Related Melanoma Small Extracellular Vesicle (sEV) mRNA Content Using Primary Melanocyte sEVs as a Reference. Int J Mol Sci. 2019;20(5). 10.3390/ijms20051235 PMC6429302 30870978

[35] Li J, Chen J, Wang S, Li P, Zheng C, Zhou X, et al Blockage of transferred exosome-shuttled miR-494 inhibits melanoma growth and metastasis. J Cell Physiol. 2019. 10.1002/jcp.28234 30723916

[36] Thomou T, Mori MA, Dreyfuss JM, Konishi M, Sakaguchi M, Wolfrum C, et al Adipose-derived circulating miRNAs regulate gene expression in other tissues. Nature. 2017;542(7642):450–5. 10.1038/nature21365 28199304 PMC5330251

[37] Fattore L, Ruggiero CF, Pisanu ME, Liguoro D, Cerri A, Costantini S, et al Reprogramming miRNAs global expression orchestrates development of drug resistance in BRAF mutated melanoma. Cell Death Differ. 2019;26(7):1267–82. 10.1038/s41418-018-0205-5 30254376 PMC6748102

[38] Vignard V, Labbé M, Marec N, André-Grégoire G, Jouand N, Fonteneau JF, et al MicroRNAs in Tumor Exosomes Drive Immune Escape in Melanoma. Cancer Immunol Res. 2020;8(2):255–67. 10.1158/2326-6066.CIR-19-0522 31857348

[39] Tang Y, Zhao Y, Song X, Song X, Niu L, Xie L. Tumor-derived exosomal miRNA-320d as a biomarker for metastatic colorectal cancer. J Clin Lab Anal. 2019;33(9):e23004 10.1002/jcla.23004 31420913 PMC6868417

[40] Zheng Y, Song A, Zhou Y, Zhong Y, Zhang W, Wang C, et al Identification of extracellular vesicles-transported miRNAs in Erlotinib-resistant head and neck squamous cell carcinoma. J Cell Commun Signal. 2020. 10.1007/s12079-020-00546-7 PMC7642164 32157550

