# Supplementary material for: Ultraviolet B irradiation enhances the secretion of exosomes by human primary melanocytes and changes their exosomal miRNA profile
Source: PLoS One. 2020 Aug 12;15(8):e0237023. doi: 10.1371/journal.pone.0237023 (PMC7423116; doi:10.1371/journal.pone.0237023)
Supplement: S1 Raw images — (PDF) [file pone.0237023.s001.pdf]

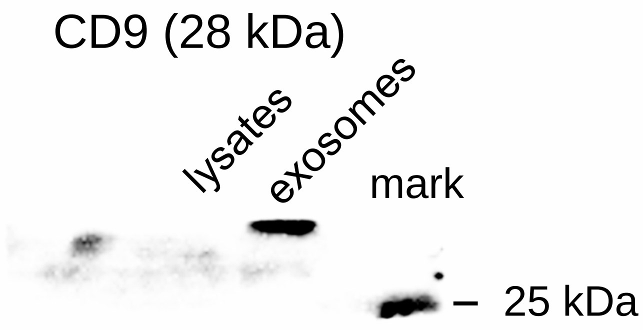

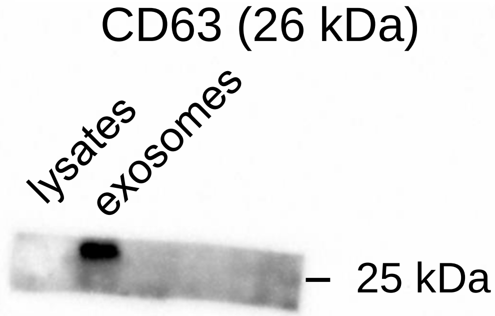

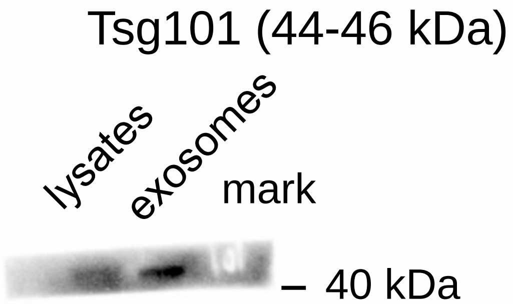

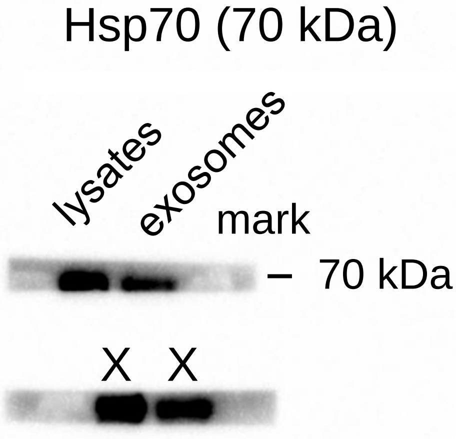

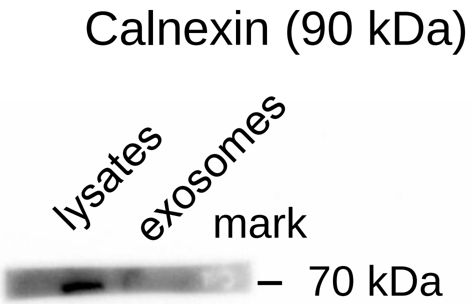

All images were capture by an optical luminescence instrument and to specify figure 1 panel.
