# Supplementary figures and images for: Ultraviolet B irradiation enhances the secretion of exosomes by human primary melanocytes and changes their exosomal miRNA profile
Source: PLoS One. 2020 Aug 12;15(8):e0237023. doi: 10.1371/journal.pone.0237023 (PMC7423116; doi:10.1371/journal.pone.0237023)

Relative mirRNA expression

□ M  
■ UVBM

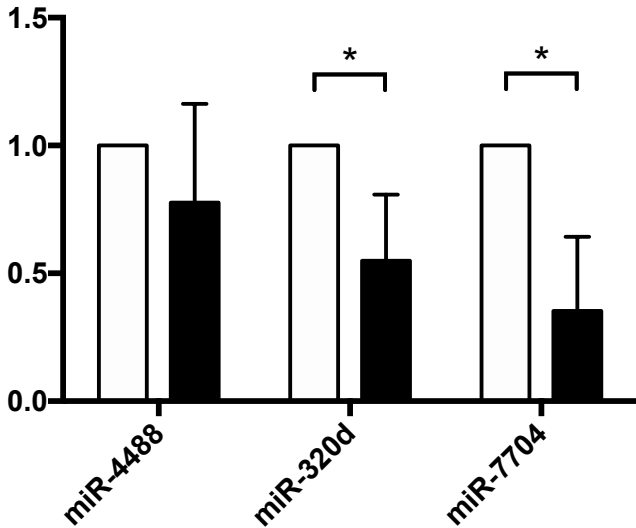

Supplement: S1 Fig — The data are from three independent experiments in each group (*, P < 0.05). (PDF) [file pone.0237023.s002.pdf]
